# Risk of Cancer in Diabetes: The Effect of Metformin

**DOI:** 10.1155/2013/636927

**Published:** 2013-09-24

**Authors:** Mojtaba Malek, Rokhsareh Aghili, Zahra Emami, Mohammad E. Khamseh

**Affiliations:** Endocrine Research Center (Firouzgar), Institute of Endocrinology and Metabolism, Tehran University of Medical Sciences, Tehran 15937-48711, Iran

## Abstract

Cancer is the second cause of death. Association of diabetes as a growing and costly disease with cancer is a major health concern. Meanwhile, preexisting diabetes is associated with an increased risk of all-cause and cancer-specific mortalities. Presence of diabetes related comorbidities, poorer response to cancer treatment, and excess mortality related to diabetes are among the most important explanations. Although diabetes appear to be a risk factor for cancer and is associated with the mortality risk in cancer patients, several factors such as diabetes duration, multiple drug therapy, and the presence of diabetes comorbidities make the assessment of the effect of diabetes treatment on cancer risk and mortality difficult. Metformin is the drug of choice for the treatment of type 2 diabetes. The available evidence from basic science, clinical, and population-based research supports the anticancer effect of metformin. However, randomized controlled clinical trials do not provide enough evidence for a strong protective effect of metformin on cancer incidence or mortality. One of the most important limitations of these trials is the short duration of the followup. Further long-term randomized controlled clinical trials specifically designed to determine metformin effect on cancer risk are needed to provide the best answer to this challenge.

## 1. Diabetes and Cancer 

The prevalence of diabetes in newly diagnosed cancer patients is reported to be from 8% to 18% [1]. A meta-analysis of 12 cohort studies reported a significant pooled adjusted risk ratio (RR) of all-cancer incidence (RR = 1.10, 95% CI, and 1.04 to 1.17). The increased risk was observed for both men (RR = 1.14, CI, and 1.06 to 1.23) and women (RR = 1.18, CI, and 1.08 to 1.28) [2].

An increase for site-specific cancer incidence has also been described in many systematic reviews and meta-analyses. Deng et al. reported a 26% increase in the incidence of colorectal cancer. The rate was similar in both women and men. These results were obtained from eight case-control and 16 cohort studies without heterogeneity between studies (*P* = 0.296) [3]. Parallel to these results, Jiang et al. also described an increased incidence of colorectal cancer in a systematic review of 41 cohort studies (summary of relative risk 1.27, 95% CI: 1.21–1.34) [4]. Although the studies showed significant heterogeneity (*P* = 0.002, *I* ^2^ = 48.4%), the incidence seems not to be affected by sex (RR = 1.25, 95% CI: 1.19–1.14 in men, and RR = 1.34, 95% CI: 1.22–1.47 in women) [5].

Breast cancer has also been shown to be more prevalent in women with type 2 diabetes (SRR = 1.27, 95% CI: 1.16–1.39). In contrast, type 1 diabetes seems not to be associated with the risk of breast cancer (SRR 1.00, 95% CI: 0.74–1.3). In type 2 diabetes, the risk was lower in prospective (23%) than in retrospective studies (36%). Adjustment for BMI showed a lower risk (16%), compared with nonadjustment (33%) [6].

Another meta-analysis included 38 studies investigating breast cancer incidence in women, and 5 studies investigating the incidence in men found a significantly increased risk in both women (RR 1.24, 95% CI: 1.15–1.35) and men (RR 1.40, 95% CI: 1.10–1.79) [7]. 

Two meta-analyses explored the incidence of gastric cancer in people with type 2 diabetes in case-control and cohort studies [8, 9]. A slight increase in the incidence of gastric cancer was reported, although the risk seems to be affected by sex. Women with diabetes showed an 18% increase in risk compared with nondiabetic individuals, while the risk of gastric cancer was similar for diabetic men compared to nondiabetic men [8].

Stevens et al. conducted a systematic review of three cohort and six case-control studies and reported pancreatic cancer by diabetes subtype. The overall RR for pancreatic cancer in type 1 or young-onset diabetes was significantly higher compared to those with no diabetes (RR 2.00, 95% CI: 1.37–3.01) [10]. Furthermore, in a meta-analysis of 35 cohort studies, diabetes was associated with an increased risk of pancreatic cancer (SRR 1.94, 95% CI: 1.66–2.27). However, there was a significant heterogeneity among the studies (*P* < 0.001, *I* ^2^ = 93.6%) [11]. In this meta-analysis, the risk of pancreatic cancer was independent of body mass index, alcohol consumption, and smoking status. 

The risk for kidney cancer was investigated in a meta-analysis of nine cohort studies. The results showed a 42% increased risk of kidney cancer, although the studies were heterogenous (*P* < 0.001). The risk was higher in women (RR 1.70, 95% CI: 1.47–1.9) than in men (RR 1.26, 95% CI: 1.06–1.49). Adjustment for BMI lowered the association significantly (RR 1.12, 95% CI: 0.99–1.27) [12]. 

The available evidence for the risk of prostate cancer in type 2 diabetes is conflicting. A recent meta-analysis, of 29 cohorts and 16 case-control studies, examined the association between type 2 diabetes and risk of prostate cancer and showed a significant inverse association (RR 0.86, 95% CI: 0.80–0.92) [13].  Table 1 summarizes the cancer risks in diabetes.

Considering cancer mortality, preexisting diabetes is associated with the risk of all-cause and site-specific mortality [2, 4, 8, 14].

Noto et al. described in a qualitative review and meta-analysis that diabetes was associated with an increased relative risk of all-cancer mortality (RR 1.16, 95% CI: 1.03–1.30) which remained significant for both sexes in a subgroup analysis [2].

Site-specific cancer mortality for colorectal cancer was evaluated in a systematic review and a meta-analysis of cohort studies by Jiang et al. They reported that diabetes was associated with a 20% increase in the incidence of colorectal cancer that was independent of sex, body mass index, family history of colorectal cancer, smoking, geographic location, and physical activity [4].

Similarly, mortality from gastric cancer in a patient with diabetes was reported to be higher than those individuals without diabetes (SRR 1.29, 95% CI: 1.04–1.59) [8]. 

Breast cancer outcomes in people with preexisting diabetes were reported in a systematic review and a meta-analysis by Peairs et al. In this study preexisting diabetes was associated with 49% increase in all-cause mortality in patients with breast cancer. In addition, these people had a more advanced stage on presentation and experienced more toxicity from chemotherapy [14].

Table 2 summarizes the potential explanations for the observed association of diabetes and mortality risk.

## 2. Hyperinsulinemia and Cancer 

One of the most important metabolic abnormalities that might explain the relationship between type 2 diabetes and cancer is hyperinsulinemia [16].

Hyperinsulinemia is the result of insulin resistance in peripheral tissues. 

Nocturnal lipolysis plays an important role in causing insulin resistance and compensatory hyperinsulinemia [17]. Insulin can have direct and indirect effect on tumor cells. Direct mitogenic effect of insulin on tumor cells is mediated through the insulin receptors that are expressed on tumor cells. 

On the other hand, insulin, in high concentrations, is capable of activating the related growth factors receptors such as insulin-like growth factor one receptors. These receptors are also expressed on all tumor cells. 

Furthermore, insulin can stimulate the synthesis of insulin growth factor one (IGF-1) and promote steroidogenesis. 

In addition, insulin has an inhibitory effect on the expression of sex hormone binding globulins. 

So, insulin can also promote tumor cell growth indirectly through elevated levels of circulating IGF-1 and sex steroids [18]. 

In 2004, Renehan et al. presented the idea of the direct link of insulin-like growth factors (IGFs) and insulin-like growth factor binding proteins (IGFBPs) and cancer risk [19].

Some studies described the association of circulating C-peptide, as the marker of insulin level, and colorectal cancer [19–34]. Similar results have been reported for breast cancer, and it has been shown that the overall risk of breast cancer is higher in the upper levels of C-peptide.

In summary, the proposed mechanisms explaining the possible positive link between hyperinsulinemia and cancer risk are

1. direct promotion of tumor proliferation [35],
2. modulation of circulating growth factors and their binding proteins [36],
3. binding to growth factor specific receptors [37].

Hyperinsulinemia has been shown to be associated with an increased risk of cancer mortality. Cremona study described a higher risk of cancer mortality (HR = 1.62, 95% CI: 1.19–2.20; *P* < 0.002) in general and a higher risk of morality for gastrointestinal cancer (HR = 2.61, 95% CI: 1.73–3.94; *P* < 0.0001) [38]. The effect of hyperinsulinemia on cancer mortality was independent of diabetes, obesity, and the metabolic syndrome. In another study on endometrial cancer patients, hyperinsulinemia was associated with a more aggressive course in patients with well or moderately differentiated endometrial adenocarcinoma [39].

## 3. Metformin and Cancer

### 3.1. Metformin-Historical Perspective

*Galega officinalis* (French lilac or goat's rue) is the plant which biguanides are derived from. In ancient Egypt and medieval Europe, it was used for the relief of polyuria [40–42].

In the 1920s, biguanides were identified as the active ingredient of *Galega officinalis* and in the 1950s, they were developed as therapeutics. Since then, metformin, phenformin, and buformin were developed and used for treatment of type 2 diabetes [43]. The problem with the latter two, that is, phenformin and buformin, was toxicity related to lactic acidosis. Hence, they were withdrawn from the market by the 1970s. 

However, metformin proved to be safe and was recognized as one of the most effective and safe therapeutics for treatment of type 2 diabetes. In the UK, it got the approval for the treatment of hyperglycemia in 1958, and three decades later it was approved in the US.

Several years later, “a new life for an old drug” was begun [44]. The role of biguanides in metabolic immunotherapy and metabolic rehabilitation opened a new window towards the future: the use of metformin beyond diabetes [44–47].

### 3.2. Mechanism of Action of Metformin

**Figure 1:**
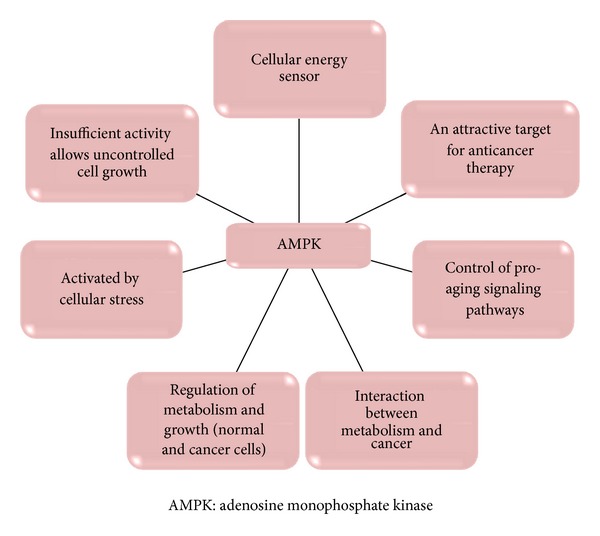
The role of AMPK* at cellular level.

Adenosine monophosphate kinase (AMPK) is the cellular energy sensor located within the cytoplasm. It is involved in regulating metabolism within the cells (Figure 1).

A protein called liver kinase B_1_ (LKB_1_) is the product of the tumor suppressor gene. Phosphorylation of AMPK catalytic subunit takes place in the presence of LKB1 and is facilitated by AMP. Increasing intracellular levels of adenosine monophosphate (AMP) activates AMPK [48, 49].

AMPK activation leads to the inhibition of mammalian target of rapamycin (mTOR) signaling, hence, downregulates gluconeogenesis by the liver.

**Figure 2:**
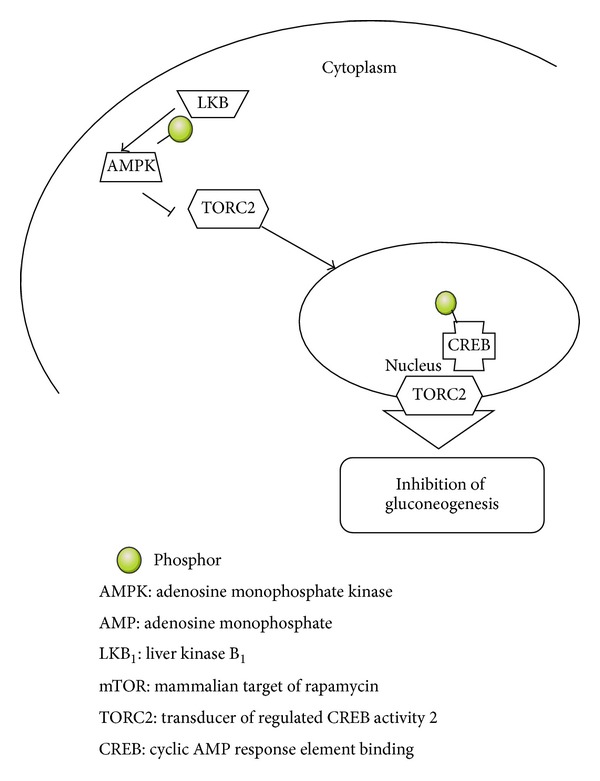
Energy sensing pathways converge on the coactivator TORC2.

Figure 2 illustrates that the energy sensing pathways converge on the coactivator TORC2.

The role of AMPK in regulating energy balance is illustrated in Table 3.

Metformin exerts both indirect (insulin dependent) and direct (insulin independent) actions at the cellular level. Its direct effect is mediated via AMPK activation and reduction of mTOR signaling pathway which leads to inhibition of gluconeogenesis in the liver, protein synthesis and cell proliferation in cancer cells [50–53].

The indirect effects of metformin are mediated through its blood glucose lowering ability and subsequent reduction of the circulating insulin level.

Metformin activates AMPK in the liver and skeletal muscles. In this way, it reduces gluconeogenesis in the liver and fosters glucose uptake by the peripheral tissues, leading to a lower blood glucose and insulin levels [40, 54].

Insulin has been known to have a mitogenic activity and theoretically could have a promoting effect on the growth of tumor cells [55, 56].

Although various anticancer effects for metformin have been described [57, 58], the LKB_1_ medicated mTOR signaling suppression seems to be the fundamental mechanism of anticancer effect of metformin.  Table 4 shows the possible anticancerogenic effects of metformin.

### 3.3. Metformin, Cancer Risk, and Cancer Mortality

Metformin is the drug of choice for the treatment of type 2 diabetes [59].

It is a safe drug, and its cost is very low. So, it remains one of the most common prescribed drugs worldwide [60]. In addition, metformin can inhibit cancer cell growth in vitro [60–62].

Hirsch et al. [63] explained that metformin selectively kills cancer stem cells and blocks tumor growth. They also observed a synergistic action of metformin with chemotherapeutic drugs in order to reduce tumor mass and prolongation of remission in nude mice. 

They showed that a combination treatment (metformin plus doxorubicin) can induce remission and maintained it for at least 60 days after treatment withdrawal. Relapse of the tumor growth was observed after 20 days in mice treated with doxorubicin alone. On the other hand, combination therapy was associated with a prolong remission that might represents a cure. 

Meta-analyses of metformin and cancer risk in diabetic patients described around one-third reduction in overall cancer risk and cancer mortality in metformin users compared with other antidiabetic drugs [14, 64].

Moreover, the pooled risk ratios for the incidence of specific cancer sites were lower in metformin users: 0.68 (95% CI, 0.53–0.88) for colorectal cancer, 0.2 (95% CI, 0.07–0.59) for hepatocellular cancer, and 0.67 (95% CI, 0.45–0.99) for lung cancer [14].

The potential role of metformin in chemoprevention for liver cancer is clearly described in a recent meta-analysis [65]. The study indicated that among patients with type 2 diabetes, metformin is associated with a 62% reduction in the estimated risk of liver cancer and 70% risk reduction for hepatocellular carcinoma (HCC). 

The finding that the risk reduction for cancer incidence varies among certain sites might be due to the difference in carcinogenesis at different sites. Growth of the epithelial malignant tumors such as colon, pancreas, and breast is affected by hyperglycemia and hyperinsulinemia [66–70]. These findings support the anticancer effect of metformin especially because the analysis was based on large population-based data obtained from multiple nations, including Asians who are generally lean and insulinopenic [15].

Although meta-analyses of observational studies suggest a cancer risk reduction around one third in metformin users, results of a recently published systematic review and collaborative meta-analyses of randomized clinical trials do not support this concept [71]. This meta-analysis obtained the data for cancer incidence from 11 RCTs and for all-cause mortality from 13 RCTs. The summary RR for the incident of cancer in people randomized to metformin compared with comparators was 1.03 (95% CI: 0.82–1.28, *I* ^2^ = 15%).

In addition, the summary RR for all-cause mortality in metformin users compared with comparators was 0.94 (95% CI: 0.79–1.12).

The analyses of the trials comparing metformin to placebo or usual care and trials with periods longer than one year were not in favor of metformin. However, the confidence intervals were wide, and there was a high clinical heterogeneity between the trials considering the comparators. Furthermore, there was not sufficient data to examine individual cancer endpoints. Another important limitation was the short follow-up period (average 4.1 years). In most observational studies, the beneficial effect of metformin to reduce the cancer risk was evident when the drug was used for more than five years [64].

As most patients with diabetes need multiple drugs for optimal metabolic control [72], the possibility of drug interactions is an important limitation for the evaluation of cancer risk of specific glucose lowering drugs in both observational studies and clinical trials. 

### 3.4. Glucose Lowering Drugs, Cancer Risk, and Cancer Mortality

Although diabetes appears to be a risk factor for some types of cancer and is associated with the risk of mortality in cancer patients, the impact of intensive glycemic control on the cancer risk reduction and improving cancer outcomes is controversial.

The majority of the evidence for the association between glycemic control and cancer outcomes is based on the epidemiologic studies which harbor considerable confounds [73–76].

Several factors such as the duration of diabetes, the need for combination drug therapy to reach metabolic targets, and the presence of diabetes comorbidities make the assessment of the effect of diabetes treatment on cancer risk and cancer mortality difficult. 

One clinical retrospective study examined the pathologic complete response rate (PCR) in women with early stage breast cancer and compared the PCR rate in diabetic women receiving metformin with non-metformin users and nondiabetic controls. The PCR was 24% in the metformin group, 8.0% in the nonmetformin group, and 16% in nondiabetic group [77, 78]. Other studies found similar effects for metformin among patients with nonsmall lung cancer and colorectal cancer with diabetes [79, 80]. However, there was no significant improvement in estimated 3-year relapse-free survival rate.

In a case control study [81], metformin was found to be associated with a risk reduction for cancer in general. UKPDS metformin study compared metformin-based intensified treatment against dietary management in overweight people with type 2 diabetes. It supports the beneficial effect of metformin on cancer mortality [82]. However, the sample size was small in this study.

An epidemiologic study found that metformin use was associated with a lower risk of cancer-related mortality compared to sulfonylureas users [61]. A meta-analysis of seventeen observational studies investigated the risk of all cancer and site-specific cancer in people with type 2 diabetes and compared metformin with sulfonylurea [83]. The meta-analysis showed that metformin was significantly associated with decreased RR for all cancer (SRR 0.61, 95% CI: 0.54–0.7), although the studies included in the final analysis were significantly heterogeneous. Furthermore, except for colorectal cancer, metformin was not associated with any significant effect on the incidence of other cancers, for example, prostate and breast cancers. 

In another study, Currie described the cancer risk according to treatment of type 2 diabetes. It was nadir for metformin monotherapy compared to sulfonylurea monotherapy, sulfonylurea plus metformin, and insulin-based therapy. The risk for insulin secretagogues was similar to that of insulin [84]. However, it should be mentioned that patients on combination or insulin therapies might be expected to have diabetes for a longer period of time. 

In a recent large population-based study [85], a lower risk of cancer in general and specific cancers was described in patients treated with metformin in comparison with those received sulfonylurea derivatives. Both groups had a similar duration of diabetes; however, the cause of death was not identified. So, they could not be able to compare the association of the risk of cancer death between metformin users and those who had been treated with sulfonylurea derivatives. 

The evidence from randomized trials differs from those based on observational studies. Home et al. [86] collected data for malignancies as an adverse event from a Diabetes Outcome Progression Trial (ADOPT) and Rosiglitazone Evaluated for Cardiovascular Outcomes and Regulation of Glycemia in Diabetes (RECORD).

Incident cancer was considered as an adverse event in these two randomized controlled clinical trials. The results suggest that metformin has no advantage over rosiglitazone for cancer protection, although there is a possibility that sulfonylureas may have a small disadvantage over the other two drugs. However, it should be emphasized that the number of malignancies was limited in these trials, and the use of insulin for long-term glucose control might dilute the differences between the medications. 

In conclusion, metformin is a safe and effective drug for treatment of type 2 diabetes with a low cost. The current evidence is not sufficient to support the anticancer effect of metformin. The protective effect of metformin might be evident over a longer period of time or in certain treatment groups, for example, insulin users.

Long-term randomized clinical trials specifically designed to determine metformin effect on cancer risk are needed to examine the hypothesis that metformin has an anticancer effect.

## Key Messages

- Type 2 diabetes is associated with an increased risk of incident cancer.
- Type 1 diabetes is associated with pancreatic cancer.
- Preexisting diabetes is associated with the risk of all-cause and cancer-specific mortality.
- Available evidence from observational studies supports the hypothesis that metformin has an anticancer effect.
- Randomized controlled clinical trials do not provide strong evidence supporting the protective effect of metformin on cancer incidence and mortality.
- Long-term RCTs are needed to specifically target the effect of metformin on cancer risk and cancer mortality.

## Figures and Tables

**Table 1:** Cancer risks in diabetes.

First author, year of publication	Study method [reference]	Sample	Risk of cancer among DM participants (95% CI)
Noto, 2011	Meta-analysis [2]	12 cohort studies	Men RR = 1.14 (1.06–1.23) Women RR = 1.18 (1.08–1.28)
Deng, 2012	Systematic review and meta-analysis [3]	8 case-control studies 16 cohort studies	RR = 1.26 (1.20–1.31)
Jiang, 2011	Systematic review and meta-analysis [4]	30 cohort studies	Summary RR = 1.27 (1.21–1.34)
Krämer, 2012	Meta-analysis [5]	8 case-control studies 21 cohort studies	Men RR = 1.29 (1.19–1.140) Women RR = 1.34 (1.22–1.47).
Boyle, 2012	Meta-analysis [6]		Summary RR = 1.27 (1.16–1.39)
Hardefeldt, 2012	Meta-analysis [7]	43 studies	Men RR = 1.40 (1.10–1.79) Women RR = 1.24 (1.15–1.35)
Tian, 2012	Meta-analysis [8]	7 case-control studies 18 cohort studies	Summary RR = 1.11 (1.00–1.24)
Ge, 2011	Meta-analysis [9]	4 case-control studies 17 cohort studies	Summary RR = 1.09 (0.98–1.22) Women Summary RR = 1.18 (1.01–1.39)
Stevens, 2007	Systematic review and meta-analysis [10]	6 case-control studies 3 cohort studies	RR = 2.00 (1.37–3.01)
Ben, 2011	Meta-analysis [11]	35 cohort studies	Summary RR = 1.94 (1.66–2.27)
Larsson, 2011	Meta-analysis [12]	9 cohort studies	Men RR = 1.26 (1.06–1.49) Women RR = 1.70 (1.47–1.97)
Bansal, 2013	Meta-analysis [13]	16 case-control studies 29 cohort studies	RR = 0.86 (0.80–0.92)
Peairs, 2011	Systematic review and meta-analysis [14]	8 studies	HR = 1.49 (1.35–1.65)
Noto, 2010	Systematic review and meta-analysis [15]	1 case-control study 4 cohort studies	Men RR = 1.25 (1.06–1.46) Women RR = 1.23 (0.97–1.56)

**Table 2:** The potential explanations for the associations between the risk of all-cause and site-specific mortality in diabetes.

(1) Increase in cell proliferation rate in the presence of hyperglycemia and hyperinsulinemia	
(2) Increase in cell permeability and changes in basement membranes caused by an increased level of reactive oxygen species (ROS)	
(3) Presence of diabetes related morbidities which may alter clinical decisions regarding the treatment for the cancer	
(4) Poorer response to the cancer treatment	
(5) Suboptimal cancer screening programs in diabetic patients	
(6) Excess mortality risk related to diabetes	

**Table 3:** Role of AMPK in regulating energy balance at the whole body.

Pancreas	Modulation of insulin secretion
Liver	Inhibition of gluconeogenesis Inhibition of cholesterol synthesis
Adipose tissue	Inhibition of lipolysis
Skeletal muscle	Increase glucose uptake Increase fatty acid oxidation
Hypothalamus	Increase food intake
Heart	Increase glucose uptake Increase fatty acid oxidation

AMPK: adenosine monophosphate kinase.

**Table 4:** The putative anticarcinogenic effects of metformin.

(1) Activation of LKB_1_/AMPK pathway	
(2) Induction of cell cycle arrest and/or apoptosis	
(3) Inhibition of protein synthesis	
(4) Reduction in circulating insulin levels	
(5) Activation of the immune system	
(6) Eradication of cancer stem cells	
(7) Reduced IGF1, insulin, and HER2-mediated signaling	
(8) Inhibition of angiogenesis	

AMPK: adenosine monophosphate kinase.

LKB_1_: liver kinase B_1_.

IGF1: insulin-like growth factor 1.

HER2: human epidermal growth factor receptor 2.

## Abbreviations

UK: United Kingdom
US: United States
AMPK: Adenosine monophosphate kinase
LKB_1_: Liver kinase B_1_
AMP: Adenosine monophosphate
mTOR: Mammalian target of rapamycin
TORC2: Transducer of Regulated CREB activity 2
CREB: Cyclic AMP response element binding
IGF1: Insulin-like growth factor 1
HER2: Human epidermal growth factor receptor 2
IGFBPs: Insulin-like growth factor binding proteins
ROS: Reactive oxygen species
PCR: Pathologic complete response rate
UKPDS: United Kingdom Prospective Diabetes Study
EASD: European Association for the Study of Diabetes
ADA: American Diabetes Association
SRRs: Standardised registration ratios
HCC: Hepatocellular carcinoma
RRs: Relative risks.
